# Unpacking the Impact of Writing Feedback Perception on Self-Regulated Writing Ability: The Role of Writing Self-Efficacy and Self-Regulated Learning Strategies

**DOI:** 10.3390/bs15020100

**Published:** 2025-01-21

**Authors:** Soonhee Hwang

**Affiliations:** Department of Liberal Arts & Science, Hongik University, 2639, Sejong-ro, Jochiwon-eup, Sejong-si 30016, Republic of Korea; soonheehwang@hongik.ac.kr

**Keywords:** undergraduates, writing achievement, self-regulated writing ability, writing feedback perception, writing self-efficacy, self-regulated learning strategies

## Abstract

Writing is a goal-oriented cognitive activity that requires metacognition and is essential for learners across all academic levels. However, many students struggle with writing skills, which can negatively affect their academic success and future opportunities. This study aimed to analyze factors influencing undergraduates’ writing achievement and to explore the relationships among writing feedback perception, writing self-efficacy, self-regulated learning strategies, and self-regulated writing ability. The study was conducted in two parts: Phase 1 examined the factors affecting the writing achievement of 196 undergraduates, while Phase 2 explored the mediating role of writing self-efficacy and self-regulated learning strategies in the relationship between the writing feedback perception and self-regulated writing ability of 306 undergraduates in Korea. Data were analyzed using Pearson’s correlation, regression, and multiple mediation analyses. Findings from Phase 1 showed that self-regulated learning strategies and self-regulated writing ability predicted writing achievement, while writing feedback perception and writing self-efficacy did not. In Phase 2, three key results emerged. First, writing feedback perception was a significant predictor of writing self-efficacy and self-regulated learning strategies. Second, writing feedback perception, writing self-efficacy, and self-regulated learning strategies significantly predicted self-regulated writing ability. Third, writing self-efficacy and self-regulated learning strategies mediated the relationship between writing feedback perception and self-regulated writing ability. The findings contribute to a deeper understanding of the mechanisms underlying undergraduates’ writing achievement by emphasizing the indirect effects of writing feedback perception through writing self-efficacy and self-regulated learning strategies. The results underscore the role of fostering writing self-efficacy and equipping learners with effective self-regulated learning strategies to enhance writing skills. Additionally, the study highlights the value of well-designed writing feedback as a foundational element for cultivating students’ confidence and autonomy in their writing practices.

## 1. Introduction

Writing is a goal-oriented cognitive activity (J. R. Hayes, 1996) and an act of self-regulation that requires metacognition, i.e., observing one’s writing, planning, monitoring, evaluating, and regulating cognitive processes (Harris & Graham, 2016). Writing is not only essential for communication and self-expression but it also serves as a powerful tool for learning (Graham, 2006). Writing is a critical competency for all learners, regardless of school level or major, and has recently been oriented toward writing across the curriculum (Bai et al., 2022; Klein & Boscolo, 2016; McLeod, 2020). For university students, writing is a critical academic skill as it contributes to reading comprehension, academic achievement, and overall literacy (Abdalrahman, 2021; Bangert-Drowns et al., 2004). Despite the growing emphasis on writing proficiency throughout education, learners often struggle with inadequate writing skills. For instance, American adolescents’ literacy skills, namely, their ability to read and write, are critically poor (Graham & Hebert, 2010) and insufficient for higher education. First-year university students face challenges caused by a lack of interest in writing and increased writing apprehension, as observed in general (Baez, 2005; Martinez et al., 2011) and specifically in Korea (Hwang, 2019). These issues highlight the need to explore the factors influencing writing achievement and develop strategies to improve learners’ writing outcomes.

Feedback has been identified as a significant predictor of self-regulated learning (SRL) and writing outcomes. Effective feedback not only supports cognitive development but also enhances motivational and socio-emotional aspects of learning (Hattie & Timperley, 2007). However, the understanding of learners’ perception of feedback and how it impacts their self-regulation and writing abilities, particularly in higher education, remains limited. This study aims to address these gaps by examining the factors influencing writing achievement and exploring the mediating roles of writing self-efficacy and SRL strategies in the relationship between writing feedback perception and self-regulated writing ability.

**Figure 1:**
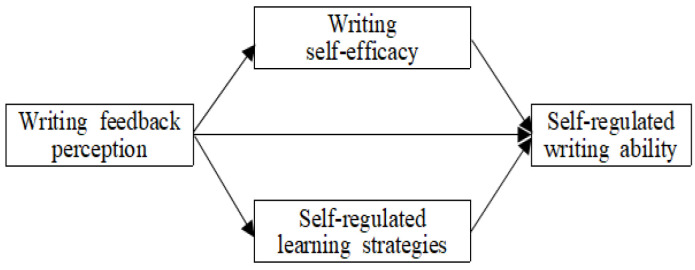
Research model.

Building on the self-regulated learning framework (Zimmerman, 2000) and the social cognitive perspective (Bandura, 1997), this study addresses two specific research objectives: Phase 1 investigates the predictors of successful writing achievement in undergraduate students, while Phase 2 explores how writing self-efficacy and SRL strategies mediate the relationship between writing feedback perception and self-regulated writing ability. To achieve the aims of this study, the research questions (RQs) and model (Figure 1) were set as follows:

1. Do undergraduate students’ writing feedback perception, writing self-efficacy, SRL strategies, and self-regulated writing ability predict their writing achievement?
2. What roles do writing self-efficacy and SRL strategies play in the relationship between undergraduate students’ writing feedback perception and self-regulated writing ability?
  2.1 Does writing feedback perception predict writing self-efficacy and SRL strategies?
  2.2 Do writing feedback perception, writing self-efficacy, and SRL strategies predict self-regulated writing ability?
  2.3 Does writing self-efficacy mediate the relationship between writing feedback perception and self-regulated writing ability?
  2.4 Do SRL strategies mediate the relationship between writing feedback perception and self-regulated writing ability?

The findings are expected to provide insights into improving undergraduate writing outcomes and inform the design of support measures in writing education.

The rest of this paper proceeds as follows: Section 2 presents the literature review and Section 3 the methodology. Section 4 outlines the data analysis and results. Section 5 discusses the findings. Section 6 concludes with a summary of key insights and recommendations for future research.

## 2. Literature Review

### 2.1. Predictors of Writing Achievement

Writing achievement is influenced by diverse factors, which can be categorized as cognitive, emotional and psychological, environmental, social, and feedback-related factors.

Cognitive factors influencing writing achievement include vocabulary ability (Olinghouse & Wilson, 2013), background knowledge (Moravcsik & Kintsch, 2013), writing self-efficacy (Pajares, 2003), SRL strategies (Boekaerts & Cascallar, 2006; Zimmerman, 2000), and self-regulated writing ability (Zhou & Hiver, 2022). Writing self-efficacy—the belief in one’s ability to succeed in writing—enhances learners’ motivation and persistence. SRL strategies improve writing outcomes by facilitating goal-setting, self-monitoring, and self-reflection. Together, these factors contribute to more effective and organized writing performance.

Emotional and psychological factors include writing motivation (Huerta et al., 2017; Wigfield & Cambria, 2010), emotional state (Pekrun et al., 2002), and writing anxiety (Cheng, 2004; Hwang, 2019). Positive emotions foster creativity and productivity, while writing anxiety and stress impair concentration and hinder writing performance. Emotional factors, such as enjoyment and perceived value, further influence engagement and persistence in writing tasks.

Environmental factors, such as physical and digital environments, resource accessibility, and broader cultural contexts, also influence writing outcomes (J. R. Hayes, 1996; Myhill et al., 2023). A suitable workspace and access to resources are crucial for productivity, while social and cultural expectations shape learners’ attitudes toward writing. Feedback-related factors (Hattie & Timperley, 2007; Shute, 2008) play a pivotal role in identifying areas for improvement, enhancing learners’ confidence, and improving writing performance (Lizzio & Wilson, 2008).

Feedback is a multidimensional activity that significantly influences writing outcomes and learners’ writing self-efficacy and strategies (Lizzio & Wilson, 2008; Yu & Schunn, 2023). It encompasses emotional, relational, and social aspects, making its perceptions and effects inherently variable depending on learners’ emotional maturity, self-efficacy, motivation (Brown et al., 2016; Marrs et al., 2016; Pitt & Norton, 2017), personal understanding, values (Li & Luca, 2014), and individual differences. From a social cognitive perspective (Bandura, 1997), feedback serves as a critical environmental factor that shapes learners’ personal cognition and learning behaviors (Zimmerman, 1989, 2000). Within the framework of SRL, feedback helps students track their progress, focus on learning objectives, and enhance their outcomes (Ozan & Kıncal, 2018). Specifically, feedback affects learners’ ability to self-regulate by influencing their cognitive, behavioral, and emotional processes (Zimmerman, 2000). Feedback is closely related to SRL and writing performance. Undergraduate students’ perception of writing feedback can be seen as an indicator of environmental influences, which interact with personal and behavioral factors in the writing process. In other words, the external environment influences personal cognition, which subsequently directs learning behavior (Schunk & DiBenedetto, 2020). However, the understanding of how students perceive feedback from instructors and its effects on their writing-related learning processes is limited (Brown et al., 2016; He et al., 2023).

Few studies have clarified the mechanisms through which feedback perceptions influence self-regulated writing (Ekholm et al., 2015; Zumbrunn et al., 2016). In this context, writing self-efficacy and SRL strategies play pivotal roles. Writing self-efficacy, a personal factor, reflects learners’ confidence in their writing abilities, while SRL strategies, as behavioral factors, involve specific actions such as goal-setting, planning, time management, and self-assessment. These factors collectively contribute to self-regulated writing ability, which enables learners to monitor progress and adjust strategies throughout the writing process. The universal relevance of feedback and SRL is highlighted in English as a Foreign Language (EFL) contexts, where studies emphasize the interplay between self-efficacy, SRL strategies, and feedback perception (Sun & Wang, 2020; Teng, 2024). These studies demonstrate how feedback can be tailored to diverse cultural and educational settings, fostering autonomy and improving writing proficiency. Such findings underscore the global importance of feedback in shaping educational practices and learning outcomes. Recent research highlights the integration of socio-emotional dimensions, such as emotion regulation and resilience, into discussions of feedback and SRL. For instance, Mastrokoukou et al. (2024) illustrate how emotion regulation mediates the relationship between resilience and psychological distress, suggesting that socio-emotional factors can influence feedback perception. Similarly, Lin et al. (2024) demonstrate how resilience and future orientation impact academic achievement through mechanisms like social support. These perspectives underscore the importance of incorporating emotion regulation and socio-emotional factors into feedback frameworks to enhance learning experiences.

Taken together, feedback has been shown to predict self-efficacy, self-regulation, and SRL outcomes, including writing performance (Ajjawi et al., 2022; Wisniewski et al., 2020). Writing feedback perception is closely related to writing self-efficacy, SRL strategies, and writing achievement (Ekholm et al., 2015; Graham et al., 2007; Magno & Amarles, 2011; Zumbrunn et al., 2016). Writing feedback perception also influences writing performance and self-regulated writing ability through the mediation of writing self-efficacy and SRL strategies (Hattie & Timperley, 2007; Wisniewski et al., 2020). By integrating socio-emotional and global dimensions, feedback can be understood within a broader theoretical framework. This approach provides a comprehensive perspective on the role of feedback in fostering SRL and writing proficiency, emphasizing its importance as both a cognitive and motivational tool. Accordingly, this study examines whether undergraduate students’ perceptions of writing feedback significantly influence self-regulated writing ability through the mediating roles of writing self-efficacy and SRL strategies.

Writing performance and achievement can be measured based on various aspects, such as content quality, text structure, paragraph organization, logical flow (D. McCutchen et al., 2009), and the writing process (J. R. Hayes & Flower, 2016). Additionally, although there are various measurement methods (Weigle, 2002), writing scores are typically calculated for evaluation purposes. For instance, holistic scoring evaluates the overall quality of a text using a single total score. Analytic scoring evaluates several aspects of writing (e.g., content, organization, grammar, vocabulary, and spelling) individually by summing the scores of each item to calculate the total score. Criterion-referenced scoring evaluates writing based on predetermined criteria or rubrics aligned with the educational objectives. Portfolio assessment involves evaluating a collection of writing that a learner has produced over a certain period and is used to comprehensively assess the learner’s development and achievements.

### 2.2. Writing Feedback Perception and Self-Regulated Writing Ability

Feedback positively impacts academic achievement, self-efficacy, self-regulation, and SRL (Brown et al., 2016; Wisniewski et al., 2020) in various learning situations, including writing. As writing is a complex cognitive activity, learners encounter various difficulties at different stages of the writing process. Numerous studies have shown that feedback is an effective means of improving learners’ writing difficulties (Lizzio & Wilson, 2008; Yu & Schunn, 2023), enhancing their motivation for writing tasks (Nicol & Macfarlane-Dick, 2006; Pajares, 2003), and improving writing self-regulation (Cleary & Zimmerman, 2004). Given that feedback encompasses emotional, relational, and social dimensions, how it is perceived, understood, and responded to can vary depending on the learner. In this context, writing feedback perception—how students interpret feedback from instructors or peers on their writing—can range from “very positive” to “very negative” and includes learners’ emotional reactions and openness to feedback (Zumbrunn et al., 2016). For example, perception of writing feedback may differ between high and low achievers in writing, as well as between learners with high and low writing self-efficacy.

Self-efficacy is a key variable mediating self-regulation, feedback perception, and achievement (Brown et al., 2016), and feedback perception can vary according to learners’ level of self-efficacy. In other words, learners with a positive perception of feedback tend to have higher self-efficacy than those with a negative perception (Caffarella & Barnett, 2000). Learners with high self-efficacy are more likely to perceive negative feedback as a “challenge” rather than a “threat” (Putwain et al., 2013), and the more useful the feedback is perceived to be, the higher their academic achievement (Lilley & Barker, 2007). In this context, the perception of writing feedback is closely related to writing performance, grades (Hillocks, 2009; A. L. McGrath et al., 2011), and motivation (Caffarella & Barnett, 2000). College students who perceive writing feedback as useful tend to achieve better writing grades than those who do not (A. L. McGrath et al., 2011). Learners who view critical feedback on writing constructively and positively tend to have more positive perceptions and evaluations of their writing abilities, leading to higher writing self-efficacy. Numerous studies have found that learners’ writing feedback perception is closely related to self-efficacy, motivation, self-regulation, and achievement in writing (Ekholm et al., 2015; Evans, 2013; Graham et al., 2007; Magno & Amarles, 2011; Zumbrunn et al., 2016). Perception of writing feedback has been reported to mediate the relationship between writing self-efficacy and self-regulation (Ekholm et al., 2015; Zumbrunn et al., 2016).

In summary, because perception of writing feedback is closely related to writing self-efficacy, self-regulation, and performance, it is important to understand learners’ perspectives and perceptions of writing feedback to aid effective writing performance. However, there is insufficient research on how the perception of writing feedback influences writing self-regulation, successful performance, and achievement (Ekholm et al., 2015; Rowe, 2011). Therefore, further research is required in this area.

### 2.3. Writing Self-Efficacy, Self-Regulated Learning Strategies, and Self-Regulated Writing Ability

Writing self-efficacy is a critical variable affecting writing achievement, and extensive research has demonstrated its impact on writing success (Pajares, 2003; Schunk & Zimmerman, 2007). Writing self-efficacy is a strong predictor of writing performance, success, and outcomes (Pajares, 2003). Moreover, it is a domain-specific form of self-efficacy (Bandura, 1986) that refers to confidence in one’s ability to succeed in a particular area. Self-efficacy, in a general sense, plays an even more crucial role in cognitively complex and challenging areas, such as writing (Bruning et al., 2013). Just as self-efficacy is a major component of self-regulation (Bandura & Cervone, 1983), writing self-efficacy, which refers to confidence and belief in successful writing performance, is also a key component of writing self-regulation (Graham & Harris, 2000) and influences successful writing performance (Pajares, 2003; Schunk & Zimmerman, 2007).

It is well known that learners with high writing self-efficacy tend to be more actively engaged in writing activities, put more effort into writing tasks, set higher goals, and, consequently, are more successful in their writing performance than their peers with lower writing self-efficacy (García-Sánchez & Fidalgo-Redondo, 2006; Schunk & Zimmerman, 2007). Writing self-efficacy is closely related to various factors associated with writing performance, such as metacognitive strategies (Brunstein & Glaser, 2011; Hwang, 2023); self-regulated writing ability; perception of feedback (Ekholm et al., 2015; Zumbrunn et al., 2016); and writing apprehension (Hidi & Boscolo, 2006; Hwang, 2023). As mentioned previously, learners’ writing difficulties are closely related to self-efficacy and self-regulation (Graham & Harris, 2003). Writing self-regulation involves actions related to planning, goal-setting, strategy use, emotional control, evaluation, and revision.

Specifically, self-regulation strategies used during the writing process include planning, goal-setting, self-monitoring, self-instruction, revision, and seeking help (Zimmerman, 2000). These strategies help maintain focus on the task and are essential for the development of writing skills (García-Sánchez & Fidalgo-Redondo, 2006). Numerous previous studies have reported a positive correlation between writing self-efficacy and self-regulation in writing (Pajares, 2003; Pintrich & De Groot, 1990; Schunk & Zimmerman, 2007; Zhou & Hiver, 2022). For instance, higher writing self-efficacy in middle-school students is associated with higher levels of self-regulatory behaviors in writing (Pintrich & De Groot, 1990). Additionally, there is a correlation among writing self-efficacy, self-regulation, and achievement among college students, and writing self-efficacy and self-regulated writing ability have been shown to predict grades in writing courses (Zhou & Hiver, 2022).

SRL strategies are representative variables that impact academic achievement across various learning environments and contexts (Pintrich & De Groot, 1990), including writing. Studies addressing the relationship between SRL strategies and self-regulated writing abilities (Bruning & Horn, 2000; Graham et al., 2005; Teng & Zhang, 2016; Zimmerman & Risemberg, 1997) have explored how learners use SRL strategies in writing performance and processes, and how these strategies impact writing outcomes. For example, Zimmerman and Risemberg (Zimmerman & Risemberg, 1997) analyzed the impact of SRL strategies on writing self-regulation abilities and found that learners who used these strategies exhibited better self-regulated writing abilities. Notably, self-evaluation and feedback utilization played important roles. Bruning and Horn (Bruning & Horn, 2000) examined the relationship between university students’ writing self-regulation abilities and their use of SRL strategies. The study found that students who used these strategies effectively (e.g., planning, self-evaluation, and feedback utilization) demonstrated higher writing self-regulation abilities, which positively impacted writing performance. These studies consistently found that learners who used SRL strategies exhibited better self-regulated writing abilities, leading to improved writing performance. As such, SRL strategies play a crucial role in helping learners set goals, monitor progress, and self-evaluate and revise during the writing process. These abilities contribute to improved writing performance. These findings suggest the need to reinforce self-regulation strategies when students complete writing tasks in educational settings.

### 2.4. Relationships Among Writing Feedback Perception, Writing Self-Efficacy, Self-Regulated Learning Strategies, and Self-Regulated Writing Ability

According to previous studies, there is a potential connection between writing feedback and self-efficacy. According to the feedback intervention (Ajjawi et al., 2022; Hattie & Timperley, 2007) and social cognitive (Bandura, 1986) theories, feedback is a key factor affecting self-efficacy, with positive and constructive feedback playing a role in boosting individuals’ self-efficacy. Numerous studies argue that effective feedback, particularly when perceived positively and constructively, enhances self-efficacy and improves learning outcomes. Specifically, when feedback is perceived as positive and constructive, it can enhance learners’ self-confidence and beliefs about their abilities—namely, self-efficacy.

In the writing context, learners who view instructor feedback positively tend to have higher self-efficacy than those who view feedback negatively (Caffarella & Barnett, 2000). Positive and useful feedback enhances self-efficacy, interest in the subject, and academic achievement (Lilley & Barker, 2007). Conversely, negative perceptions of feedback lead to lower self-efficacy, reduced confidence, and poorer writing performance (Marrs et al., 2016). Feedback is crucial for SRL, but little is known about how feedback perception impacts self-regulation during the learning process (He et al., 2023). He et al. found that learners’ perceptions of instructors’ feedback positively predicted SRL, with self-efficacy mediating this effect (He et al., 2023). This highlights the importance of feedback and supports the social cognitive theory’s view of the interaction of environmental, personal, and behavioral factors. Similarly, the perception of writing feedback significantly impacts how learners develop and apply SRL strategies, with positive feedback enhancing strategy adjustment and motivation, leading to better writing outcomes.

Regarding the relationships between writing feedback perception, writing self-efficacy, SRL strategies, and self-regulated writing ability, the impact of feedback on learners’ self-efficacy has been studied, and positive feedback has been reported to enhance self-efficacy (Shute, 2008). Another study (Ekholm et al., 2015) analyzed the relationships between perceptions of writing feedback, self-efficacy, and self-regulation beliefs (beliefs about managing the writing process strategically) and found that perceptions of writing feedback partially mediated the relationship between writing self-efficacy and self-regulation beliefs. Additionally, perceptions of feedback have been shown to positively impact attitudes toward classes and achievement through mediating effects on self-efficacy and interest, suggesting that feedback perception can be understood as a precursor to self-efficacy (Ajjawi et al., 2022; Hattie & Timperley, 2007). However, some studies have shown different results; perceptions of writing feedback mediate the relationship between writing self-efficacy and self-regulated writing ability (Ekholm et al., 2015; Zumbrunn et al., 2016), indicating that writing self-efficacy can be understood as a precursor to perceptions of writing feedback. Thus, although there is a close relationship between perceptions of writing feedback and writing self-efficacy, there is a lack of consistent findings on the precursor factor.

In summary, the evidence suggests a relationship between writing feedback perception, writing self-efficacy, SRL strategies, and self-regulated writing ability, which results in positive outcomes. Based on this rationale, the current study aimed to explore how writing feedback perception, writing self-efficacy, and SRL strategies impact self-regulated writing ability through this sequential relationship. This study tested the hypothesis of multiple mediation effects involving writing feedback perception, writing self-efficacy, SRL strategies, and self-regulated writing ability.

## 3. Methods

### 3.1. Participants and Data

To investigate factors affecting writing achievement and the relationships among writing feedback perception, SRL strategies, writing self-efficacy, and self-regulated writing ability, the researcher, as the instructor of the “Logical Thinking and Writing” course (a mandatory liberal arts course) at H University in South Korea, verbally informed all students about the purpose of the survey and the intended use of the data (course improvement and research purposes). Students who voluntarily expressed their willingness to participate in the survey provided informed consent and were included in the survey.

The sample was drawn from first-year students enrolled in the course during the specified semesters. To enhance the representativeness of the population, all enrolled students were invited to participate. While no stratified sampling techniques were employed in this study, future research could benefit from incorporating such methods to account for variables like academic performance, socioeconomic background, and linguistic proficiency. Demographic data, including participants’ academic major, year of study, and gender, were collected to address potential variability and enable subgroup analyses.

#### 3.1.1. Data Collection Procedures

Data were collected through online surveys for both Phases 1 and 2. Participants were briefed on the study procedures, including the voluntary nature of their participation, confidentiality, and the purpose of the research. Surveys were administered online using a secure platform, with clear instructions provided to minimize potential distractions or misunderstandings. Measures such as time restrictions and mandatory questions were implemented to ensure data integrity. The surveys were conducted according to the following schedule: Phase 1: The online survey was administered from 5 to 15 March 2023, prior to the start of the course. This survey measured writing feedback perception, SRL strategies, writing self-efficacy, and self-regulated writing ability. To ensure data integrity, participants were required to complete the survey within the specified time frame. After completing the 15-week course, students submitted an academic report, which was used for the final analysis of their writing achievement. Phase 2: The online survey for Phase 2 was administered from 15 to 30 September 2023, prior to the start of the course, using the same set of measures as in Phase 1. The data collection protocol prioritized compliance and validity. Participants who did not complete the surveys within the specified period were excluded from the final analysis to minimize inconsistencies. Additionally, measures were taken to verify the completeness of responses, resulting in a 100% valid response rate for both phases. To address potential selection bias, demographic data were collected to evaluate the composition of the study sample. Although the sample consisted solely of students enrolled in the “Logical Thinking and Writing” course, these students represented a diverse range of academic disciplines, which mitigated, to some extent, concerns about population representativeness.

#### 3.1.2. Phase 1

Phase 1 included 196 first-year students enrolled in the “Logical Thinking and Writing” course during the first semester of 2023 at H University. The course aimed to teach academic writing methods, culminating in the completion of academic papers. Data were collected through a pre-course survey, followed by an analysis of academic reports submitted at the end of the semester. Demographic characteristics such as major, gender, and year were recorded to evaluate sample composition.

#### 3.1.3. Phase 2

Phase 2 involved 306 first-year students enrolled in the same course during the second semester of 2023. The data collection process was identical to Phase 1, with pre-course surveys measuring the same variables to address RQs 2.1, 2.2, and 2.3.

#### 3.1.4. Sampling Strategy and Criteria

Both studies utilized random sampling, inviting students enrolled in the “Logical Thinking and Writing” course to participate. This method reduced selection bias; however, the course-specific context and characteristics of the participant population may limit the generalizability of the findings. Future studies could consider employing stratified sampling to enhance representativeness by accounting for such variables as academic performance, major, or other relevant factors. Exclusion criteria were applied to students who either did not complete the survey or were not officially enrolled in the course.

A convenience sampling method was applied, and all students enrolled in the course were invited to participate. Although this approach ensured inclusivity, it did not employ randomization or stratification to account for variability in demographic or academic characteristics. To address these limitations, future studies could implement stratified sampling strategies to enhance representativeness. Exclusion criteria included students who did not complete the surveys or were not officially enrolled in the course.

#### 3.1.5. Power Analysis

A post hoc power analysis was conducted to assess whether the actual sample sizes were sufficient to detect significant effects. The final sample sizes were 196 participants for Phase 1 and 306 participants for Phase 2. With an alpha level of 0.05 and statistical power of 0.80, these sample sizes were deemed adequate for detecting medium-to-large effect sizes in both phases.

Table 1 presents the distribution of research participants by sex and major. Participants in Phase 1 ranged in age from 19 to 22 years, with a mean age of 19.8 years (SD = 0.5). Participants in Phase 2 ranged in age from 19 to 21 years, with a mean age of 19.6 years (SD = 0.5). All participants were freshmen enrolled in “Logical Thinking and Writing” as a mandatory course, which was offered across multiple disciplines. The participants were native Korean speakers in the process of developing fundamental academic writing skills through this course.

### 3.2. Measures

The instruments used in this study were carefully selected based on their relevance and prior validation in similar contexts. Each instrument was selected and adapted to align with the research objectives. To ensure reliability and validity, Cronbach’s alpha for internal consistency was calculated, with values ranging from 0.73 to 0.91 across the subscales, indicating good-to-high reliability. Additionally, exploratory factor analysis (EFA) and confirmatory factor analysis (CFA) were conducted to evaluate construct validity. EFA was performed to examine the factor structure of the scales. The results revealed that each instrument demonstrated an appropriate factor structure:

- Writing feedback perception: A two-factor structure explained 79.3% of the total variance, with factor loadings ranging from −0.586 to −0.003.
- Writing self-efficacy: A single-factor structure explained 60.3% of the variance, with factor loadings ranging from −0.554 to −0.702.
- Self-regulated learning strategies: A multifactorial structure explained 75.0% of the variance across two main factors, with factor loadings ranging from −0.505 to −0.367.
- Self-regulated writing ability: A multifactorial structure explained 72.5% of the variance across two main factors, with factor loadings ranging from −0.473 to −0.614.

Additionally, AMOS 20.0 was used to conduct CFA to confirm the factor structure identified through EFA. The results indicated an acceptable model fit (e.g., CFI = 0.95, RMSEA = 0.06), verifying that the scales retained appropriate factor structures. These findings confirm that the instruments were valid and reliable within the context of this study, ensuring our confidence in their ability to accurately measure the constructs of interest.

Pilot testing was conducted with a subset of 25 participants representative of the target population to assess the clarity, cultural relevance, and linguistic appropriateness of the instruments. Feedback from the pilot study informed minor modifications to improve item wording and format. This process ensured that the instruments were both comprehensible and suitable for the intended population.

#### 3.2.1. Writing Feedback Perception

To assess students’ perception of writing feedback, we adapted a five-item scale from Ekholm et al. (2015) and Zumbrunn et al. (2016), with slight modifications to fit the course context. The scale consists of three sub-factors: perceptions of instructor feedback, peer feedback, and acquaintance feedback. Sample items include “I like talking with my professors about my writing” and “I feel good about my classmates’ comments about my writing.” Responses were rated on a five-point Likert scale (1 = not at all; 5 = very much). Cronbach’s α for the original scales was reported as 0.81 (Ekholm et al., 2015) and 0.83 (Zumbrunn et al., 2016). In this study, reliability was confirmed with Cronbach’s α values of 0.727 for Phase 1 and 0.730 for Phase 2, indicating good internal consistency.

#### 3.2.2. Writing Self-Efficacy

Writing self-efficacy was measured using five items adapted from Ekholm et al. (2015) and Zumbrunn et al. (2016), restructured to align with the course objectives. The scale assessed learners’ confidence in their writing abilities, with items such as “I can think of many ideas for my writing” and “I can keep writing even when it is difficult.” Responses were rated on a five-point Likert scale (1 = not at all; 5 = very much). The original scales reported Cronbach’s α values of 0.88 (Ekholm et al., 2015) and 0.82 (Zumbrunn et al., 2016). In this study, reliability was high, with Cronbach’s α values of 0.907 in Phase 1 and 0.891 in Phase 2.

#### 3.2.3. Self-Regulated Learning Strategies

Self-regulated learning (SRL) strategies were assessed using a 14-item scale developed by Zimmerman and Martinez-Pons (1986), which measures 11 sub-dimensions: self-evaluation, organizing and transforming, goal-setting and planning, seeking information, keeping records and monitoring, environmental structuring, self-consequences, rehearsing and memorizing, seeking social assistance, reviewing records, and others. Sample items include “I check over my work to make sure I did it right” and “I start studying two weeks before exams and pace myself.” Responses were rated on a five-point Likert scale (1 = not at all; 5 = very much). The original scale reported a Cronbach’s α of 0.86 (Zimmerman & Martinez-Pons, 1986). In this study, reliability was confirmed with Cronbach’s α values of 0.780 for Phase 1 and 0.803 for Phase 2, indicating good internal consistency.

#### 3.2.4. Self-Regulated Writing Ability

To measure self-regulated writing ability, 12 items were adapted from Zumbrunn et al. (2016). The scale consists of seven sub-dimensions: goal-setting, planning, self-monitoring, attention control, emotion regulation, self-instruction, and help-seeking. Sample items include “I think about who will read my writing” and “While I write, I think about my writing goals.” Responses were rated on a five-point Likert scale (1 = not at all; 5 = very much). The original scale reported a Cronbach’s α of 0.79 (Zumbrunn et al., 2016). In this study, reliability was confirmed with Cronbach’s α values of 0.751 for Phase 1 and 0.771 for Phase 2, indicating acceptable internal consistency.

#### 3.2.5. Writing Achievement

Writing achievement was assessed using academic reports submitted by students at the end of the semester in Phase 1. These reports were evaluated using both analytical and holistic methods, based on the fulfillment of report requirements. A 100-point scale was used to score the reports. Additionally, grades were assigned to evaluate the quality of the reports, and only reports deemed successful were selected.

### 3.3. Data Analysis

The data analysis was conducted separately for Phase 1 and Phase 2, employing SPSS version 29.0 and the PROCESS Macro (V. 4.2) to address the research objectives and hypotheses.

#### 3.3.1. Phase 1

In Phase 1, first, descriptive statistics were calculated to examine the basic characteristics of the variables. The skewness and kurtosis values of all variables were inspected to confirm normality, ensuring the suitability of parametric tests. Correlation analysis was performed to explore relationships among writing feedback perception, writing self-efficacy, self-regulated learning (SRL) strategies, self-regulated writing ability, and writing achievement. Linear regression analysis was then used to identify predictors of writing achievement, including writing feedback perception, writing self-efficacy, SRL strategies, and self-regulated writing ability. Writing achievement was measured as report scores and successful writing performance (e.g., receiving an A grade or higher). Multicollinearity was assessed using variance inflation factors (VIFs), with all values below 10, indicating no significant issues. Assumptions of linearity, homoscedasticity, independence of errors, and normality of residuals were verified through diagnostic plots, including residual plots and Q-Q plots. The Durbin–Watson statistic confirmed the independence of errors. In cases where minor deviations from normality were observed, robust regression methods were applied to ensure reliable estimates. Finally, logistic regression analysis was conducted to predict successful writing performance (a binary outcome). The model’s fit was evaluated using the Hosmer–Lemeshow test, and classification accuracy was reported to confirm the model’s predictive reliability.

#### 3.3.2. Phase 2

In Phase 2, the analysis began with descriptive statistics to examine data distribution, followed by correlation analysis to investigate relationships between writing feedback perception, writing self-efficacy, SRL strategies, and self-regulated writing ability. Regression analyses were conducted to assess the effects of writing feedback perception, writing self-efficacy, and SRL strategies on self-regulated writing ability. Separate models were tested to evaluate individual predictors, while combined models examined their joint effects. Assumptions for linear regression, including linearity, homoscedasticity, and normality of residuals, were verified. Robust regression methods were employed when necessary to address minor violations of these assumptions. Mediation analysis was performed using the PROCESS Macro (Model 4) to explore the indirect effects of writing feedback perception on self-regulated writing ability through writing self-efficacy and SRL strategies. Bootstrapping with 5000 samples was employed to compute bias-corrected confidence intervals (A. F. Hayes, 2017), ensuring robustness even in the presence of assumption violations. Moderation analyses were conducted to test interaction effects between key variables, such as writing feedback perception and writing self-efficacy, to identify potential moderating influences. Significant interactions were further analyzed using moderation models to better understand their implications. Finally, sensitivity analyses were performed to confirm the robustness of the findings. These analyses varied in model specifications and tested different combinations of predictors to assess the stability of the results. This approach addressed the potential overestimation of mediation effects caused by correlations among mediators, an issue highlighted by Baron and Kenny (1986) when conducting multiple simple mediation analyses, ensuring that the reported findings were accurate and reliable.

## 4. Results

### 4.1. Descriptive Statistics and Correlation Analysis Between Variables

Table 2 presents the results of the descriptive statistics and correlation analyses of the main variables in Studies 1 and 2. The skewness of the variables ranged from 0.037 to 0.347 (Phase 1) and from 0.119 to 0.338 (Phase 2), whereas kurtosis ranged from 0.038 to 1.14 (Phase 1) and from 0.065 to 0.587 (Phase 2), indicating that all variables met the assumption of normal distribution. Furthermore, the correlation analysis revealed that all correlations between the variables were significant. In Phase 1, writing feedback perception was positively correlated with writing self-efficacy, SRL strategies, self-regulated writing ability, and report scores, with correlation coefficients ranging from 0.151 (*p* < 0.05) to 0.492 (*p* < 0.01). Writing self-efficacy was positively correlated with SRL strategies, self-regulated writing ability, and report scores, with correlation coefficients ranging from 0.184 (*p* < 0.05) to 0.533 (*p* < 0.01). SRL strategies, self-regulated writing ability, and report scores showed significant positive correlations, with coefficients ranging from 0.154 (*p* < 0.05) to 0.567 (*p* < 0.01).

In Phase 2, writing feedback perception showed significant positive correlations with writing self-efficacy, SRL strategies, and self-regulated writing ability, with correlation coefficients ranging from 0.393 to 0.510 (*p* < 0.01). Writing self-efficacy was positively correlated with SRL strategies and writing ability, with correlation coefficients ranging from 0.552 to 0.553 (*p* < 0.01). SRL strategies and writing ability were significantly and positively correlated, with a coefficient of 0.648 (*p* < 0.01).

### 4.2. Factors Affecting Writing Achievement

#### 4.2.1. Factors Affecting Report Scores

Linear regression analysis (Table 3) was conducted to examine the predictors of undergraduates’ writing achievement (report scores) using writing feedback perception, writing self-efficacy, SRL strategies, and self-regulated writing ability as independent variables. The overall regression model was statistically significant (*F* = 3.170, *p* < 0.05) and explained approximately 7% of the variance in report scores (*R*^2^ = 0.068).

Among the predictors, SRL strategies (*β* = 0.214, *t* = 2.054, *p* = 0.041) had a significant positive effect, while self-regulated writing ability (*β* = −0.222, *t* = −2.224, *p* = 0.027) showed a significant negative effect on report scores. Meanwhile, writing feedback perception (*β* = 0.055, *p* = 0.527) and writing self-efficacy (*β* = 0.147, *p* = 0.123) were not statistically significant predictors, but were retained in the model for their theoretical relevance. The multicollinearity diagnostics showed no issues, with all tolerance values being above 0.1 and VIF values below 10.

#### 4.2.2. Factors Affecting Successful Writing Achievement

Among the 196 study participants, 49 (25%) achieved successful writing (receiving an A grade or higher). The results of the logistic regression analysis of the predictive factors for successful writing performance among first-year students are presented in Table 4. The Hosmer–Lemeshow statistic for the regression model was χ^2^ = 11.465, *df* = 8, *p* = 0.177, indicating that the regression model was a good fit. SRL strategies and writing abilities were significant predictors of successful writing achievement among first-year students.

### 4.3. Verification of the Predictive Power of Writing Feedback Perception on Writing Self-Efficacy and Self-Regulated Learning Strategies

Based on Phase 1, in which SRL strategies and writing ability were identified as factors influencing writing achievement, the relationships among relevant variables were further examined. To verify whether writing feedback perception predicted writing self-efficacy and SRL strategies, a regression analysis was conducted. Table 5 summarizes the results. The model for writing self-efficacy was statistically significant (*F*(1, 304) = 69.732, *p* < 0.001) and explained 18.7% of the variance in writing self-efficacy (*R*^2^ = 0.187). Writing feedback perception significantly predicted writing self-efficacy (*β* = 0.432, *p* < 0.001). Similarly, the model for SRL strategies was statistically significant (*F*(1, 304) = 107.023, *p* < 0.001), explaining 26.0% of the variance in SRL strategies (*R*^2^ = 0.260). Writing feedback perception significantly predicted SRL strategies (*β* = 0.510, *p* < 0.001). Both tolerance (1.0) and VIF (1.0) values indicated no multicollinearity issues in these analyses.

### 4.4. Verification of the Predictive Power of Writing Feedback Perception, Writing Self-Efficacy, and Self-Regulated Learning Strategies on Self-Regulated Writing Ability

To verify whether writing feedback perception, writing self-efficacy, and SRL strategies predicted self-regulated writing ability, a multiple regression analysis was conducted. Table 6 summarizes the results. The model was statistically significant (*F*(3, 302) = 90.906, *p* < 0.001) and explained 47.5% of the variance in self-regulated writing ability (*R*^2^ = 0.475). Among the predictors, SRL strategies had the largest standardized effect (*β* = 0.482, *p* < 0.001), followed by writing self-efficacy (*β* = 0.273, *p* < 0.001). Writing feedback perception showed a small and non-significant effect (*β* = 0.029, *p* = 0.561). Variance inflation factors (VIFs) for all predictors were below the commonly accepted threshold of 10, indicating no multicollinearity issues: writing feedback perception (VIF = 1.414), writing self-efficacy (VIF = 1.507), SRL strategies (VIF = 1.658). These results suggest that SRL strategies and writing self-efficacy are critical determinants of self-regulated writing ability, while writing feedback perception has a negligible direct effect in the presence of other predictors.

In addition to the regression analysis, a structural equation modeling (SEM) analysis was performed to provide a more comprehensive understanding of the relationships among the variables, including direct and indirect effects. The structural model fit indices indicated an acceptable fit to the data: X^2^(6) = 6.0, *p* = 0.174, X^2^/df = 1.5, TLI = 0.90, CFI = 0.90, and RMSEA = 0.08. SEM results revealed that writing feedback perception significantly influenced writing self-efficacy (*β* = 0.4, *p* < 0.001) and SRL strategies (*β* = 0.51, *p* < 0.001). Both writing self-efficacy and SRL strategies significantly predicted self-regulated writing ability (writing self-efficacy: *β* = 0.25, *p* < 0.001; SRL strategies: *β* = 0.47, *p* < 0.001). The direct effect of writing feedback perception on self-regulated writing ability was negligible (*β* = 0.03, *p* = 0.56), consistent with the regression results. These findings indicate that writing feedback perception influences self-regulated writing ability primarily through indirect pathways.

### 4.5. Verification of the Multiple Mediation Effects of Writing Self-Efficacy and Self-Regulated Learning Strategies on the Relationship Between Writing Feedback Perception and Self-Regulated Writing Ability

**Figure 2:**
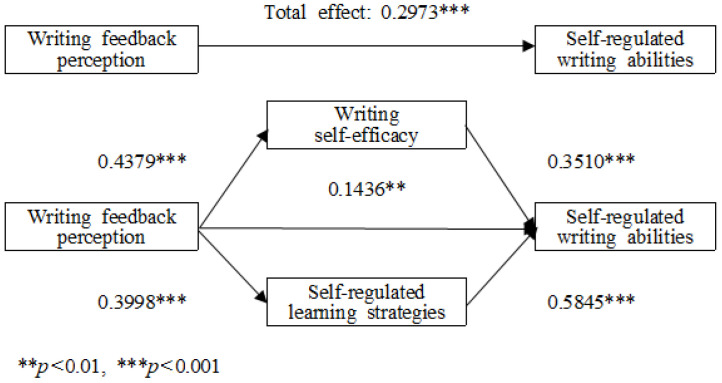
Multiple mediation effects of writing self-efficacy and self-regulated learning strategies on the relationship between writing feedback perception and self-regulated writing ability.

Building on the SEM analysis, a more detailed examination of the mediating roles of writing self-efficacy and SRL strategies was conducted using bootstrapping analysis with 5000 resamples. Figure 2 illustrates the multiple mediation model, showing the mediating effects of writing self-efficacy and SRL strategies on the relationship between writing feedback perception and self-regulated writing ability, along with unstandardized coefficient estimates.

Table 7 presents the bootstrapping results. The indirect effect of writing feedback perception on self-regulated writing ability through writing self-efficacy was significant (effect = 0.154, boot SE = 0.028, 95% CI = [0.102, 0.211]). Similarly, the indirect effect through SRL strategies was significant (effect = 0.234, boot SE = 0.036, 95% CI = [0.165, 0.304]). Neither confidence interval included 0, confirming the statistical significance of these mediation effects. Among the two mediators, SRL strategies had a stronger mediating effect than writing self-efficacy, as indicated by the larger effect size.

The direct effect of writing feedback perception on self-regulated writing ability remained non-significant in the presence of the mediators, further supporting a partial mediation model. These findings underscore the critical roles of writing self-efficacy and SRL strategies in mediating the influence of writing feedback perception on self-regulated writing ability.

## 5. Discussion

This study sought to analyze factors affecting undergraduates’ writing achievement and to explore the relationships among writing feedback perception, writing self-efficacy, SRL strategies, and self-regulated writing ability. The main findings are as follows.

### 5.1. Discussion of Findings Related to Research Questions

Phase 1 found that undergraduates’ SRL strategies and self-regulated writing ability predicted successful writing achievement (report scores and grades). By contrast, writing feedback perception and writing self-efficacy did not predict successful writing achievement in this study. These findings regarding the effects of SRL strategies and self-regulated writing ability on successful writing achievement support the results of numerous studies reporting that self-regulated writing significantly impacts writing achievement. These studies have also shown that the level of SRL strategies is closely related to writing achievement and self-regulated writing ability (Boekaerts & Cascallar, 2006; Bruning & Horn, 2000; Graham & Harris, 2003; Graham et al., 2005; Teng & Zhang, 2016; Zhou & Hiver, 2022; Zimmerman, 2000; Zimmerman & Risemberg, 1997).

Self-regulated writing ability—which encompasses the ability to plan, monitor, and evaluate the writing process—plays a crucial role in enabling more organized and effective writing. SRL strategies (e.g., goal-setting, self-monitoring, and self-reflection) are instrumental in maintaining focus and improving writing outcomes. These strategies not only predict writing achievement but also empower learners to manage and regulate their learning, which can positively impact the development of their writing skills. By setting and planning goals, monitoring progress through self-assessment, and making necessary adjustments, learners can cultivate abilities that are essential for successfully completing writing tasks. These findings imply that instructor interventions to enhance SRL strategies and writing ability are necessary for learners’ successful writing achievement and improved outcomes.

By contrast, Phase 1 showed that writing feedback perception and self-efficacy do not predict successful writing achievement, which is somewhat surprising as it contradicts numerous previous studies. Prior research has consistently shown that writing feedback perception (Ekholm et al., 2015; Magno & Amarles, 2011; Hillocks, 2009; A. L. McGrath et al., 2011) and self-efficacy (Pajares, 2003; Schunk & Zimmerman, 2007) are significant predictors of writing achievement. However, our findings are consistent with previous research that pointed out the limited impact of writing feedback perception and self-efficacy on writing achievement (Graham & Perin, 2007; Huerta et al., 2017; Troia et al., 2013). Specifically, these studies suggest that the influence of writing feedback and self-efficacy on writing achievement may be limited (Graham & Perin, 2007; Troia et al., 2013) and that high writing self-efficacy does not necessarily guarantee successful writing achievement, indicating the need to consider other factors (Huerta et al., 2017). There is a possibility of overestimating the role of writing self-efficacy in writing achievement, as learners with high writing self-efficacy may overestimate their actual achievement or neglect their writing preparation. Likewise, writing achievement is not determined by a single factor but is influenced by various factors, such as cognitive abilities, background knowledge, motivation, time management, and environment. Recent findings by Mastrokoukou et al. (2024) suggest that socio-emotional factors, such as emotion regulation, mediate feedback perception and influence its effectiveness. These perspectives highlight the complex role of feedback in writing achievement and suggest that integrating emotion regulation and socio-emotional factors into feedback frameworks could enhance learning experiences. Thus, for feedback to lead to successful writing achievement, not only is the quality, type, frequency, method, and timing of the feedback important, but so is how the learner perceives and applies it. Therefore, instructors’ efforts are needed to enhance learners’ abilities to understand and apply feedback to improve their writing skills. Situational factors related to writing tasks (e.g., the nature of the task, evaluation criteria, and instructor expectations) can also impact writing achievement. Considering these results, our finding that writing feedback perception and self-efficacy do not predict writing achievement reflects the complexity of the writing process and the various influencing factors. This suggests that, to improve writing achievement, a more multifaceted approach by instructors and personalized support for learners is necessary.

The main findings of Phase 2 indicate that the perception of writing feedback significantly predicts both writing self-efficacy and SRL strategies. These results align with prior research emphasizing the strong relationship between learners’ perceptions of writing feedback and their self-efficacy (Ekholm et al., 2015; Evans, 2013; Graham et al., 2007; Magno & Amarles, 2011; Sun & Wang, 2020; Zumbrunn et al., 2016). Similarly, studies have shown that feedback perception is closely linked to writing performance, writing grades (Graham, 2022; Hillocks, 2009; A. L. McGrath et al., 2011), and self-regulated writing abilities. Feedback that learners perceive as constructive and useful not only boosts their confidence in their writing abilities but also encourages the development of self-regulatory practices. Additionally, Lin et al. (2024) highlighted the role of resilience and socio-emotional support in academic achievement, further reinforcing the importance of positive feedback perception in enhancing learners’ self-efficacy and motivation. To maximize the impact of feedback, instructors should ensure that it is timely, specific, and aligned with clear instructional goals (Hillocks, 2009). These qualities help learners perceive feedback as actionable and valuable, thereby enhancing both their self-efficacy and self-regulatory abilities. The finding that writing feedback perception influences writing self-efficacy and self-regulation underscores the essential role of feedback throughout the writing process. When learners perceive feedback as useful, it not only improves their confidence but also strengthens their ability to self-regulate during writing tasks. For instructors, this implies the need to adopt teaching strategies that help learners effectively receive and apply feedback. Feedback delivery should focus on quality, content, and method, as these factors significantly influence learners’ perceptions and subsequent writing performance. Constructive feedback fosters greater confidence and motivation, ultimately leading to improved writing outcomes. The importance of psychological factors in writing instruction is evident, as learners who perceive feedback positively are more likely to actively incorporate it into their learning process. Thus, support mechanisms must be in place to help learners view feedback constructively and use it to enhance their writing.

In addition, writing feedback perception, writing self-efficacy, and SRL strategies were significant predictors of self-regulated writing ability. Among these predictors, writing self-efficacy played a critical role, aligning with previous studies that highlight the pivotal role of confidence in fostering self-regulation (Pajares, 2003; Pintrich & De Groot, 1990; Schunk & Zimmerman, 2007; Zhou & Hiver, 2022). Writing self-efficacy enables learners to set goals, monitor progress, and adjust their strategies, thereby enhancing their ability to regulate the writing process. Similarly, SRL strategies—such as goal-setting, planning, and self-monitoring—are integral to effective self-regulated learning and writing performance. This aligns with the findings of Zimmerman and Risemberg (1997), which emphasize the effectiveness of SRL strategies in enhancing writing abilities. Educators can support self-regulated writing by providing specific guidelines, encouraging reflective practices, and offering constructive feedback throughout the writing process (García-Sánchez & Fidalgo-Redondo, 2006). Strategies for enhancing writing self-efficacy should also include the reduction of writing anxiety through activities such as oral compositions or small group tasks, which have been shown to effectively reduce anxiety and foster confidence (De La Paz & Butler, 2018). Moreover, instructors can enhance learners’ self-efficacy by breaking down writing tasks into manageable stages and providing specific feedback at each step (García-Sánchez & Fidalgo-Redondo, 2006). Encouraging students to create writing portfolios or maintain reflective journals can help shift the focus from the final product to the process, gradually reducing writing anxiety and avoidance behaviors (De La Paz & Butler, 2018). Creating a positive emotional environment (Bruning & Horn, 2000) and recognizing students’ writing achievements (Lam & Law, 2007) are also crucial for fostering writing self-efficacy and motivation.

Finally, mediation analyses revealed that writing self-efficacy and SRL strategies mediated the relationship between writing feedback perception and self-regulated writing ability. This finding supports social cognitive theory, which posits that environmental factors (e.g., feedback perception) influence behavioral outcomes (e.g., self-regulated writing ability) through personal factors (e.g., self-efficacy) (Bandura, 1986). The mediating roles of writing self-efficacy and SRL strategies align with studies by Sun and Wang (2020) and Teng (2024), which emphasize the interplay between feedback perception, self-efficacy, and SRL strategies in EFL contexts. Additionally, Mastrokoukou et al. (2024) noted that socio-emotional factors mediate the relationship between resilience and academic outcomes, highlighting the importance of emotional support and effective feedback in reinforcing self-regulatory abilities.

Taken together, these findings underscore the importance of applying a holistic approach to writing instruction that integrates feedback, SRL strategies, and socio-emotional factors. To strengthen university students’ writing skills and improve writing outcomes, instructors must provide constructive, productive, and positive feedback to enhance learners’ perceptions of feedback, foster their confidence, and reinforce their use of effective self-regulatory strategies. By addressing both cognitive and emotional dimensions of learning—such as resilience and emotion regulation—educators can better support the development of learners’ writing abilities and overall academic success. These results also highlight the complexity of the relationships between feedback perception, self-efficacy, and SRL strategies, emphasizing that improving writing outcomes requires coordinated efforts to incorporate these elements. This study provides meaningful insights into the predictors of successful writing achievement and self-regulated writing ability, specifically by highlighting the mediating roles of writing self-efficacy and SRL strategies. Future research should further explore the interplay of these factors in diverse educational contexts to deepen our understanding of how self-regulation and feedback interact to influence writing achievement.

### 5.2. Theoretical Implications

Our findings confirmed the centrality of self-regulation theories, particularly in academic writing, in which SRL strategies and self-regulated writing ability are pivotal. SRL strategies significantly predicted both writing achievement and self-regulated writing ability, supporting Zimmerman’s (Zimmerman, 2000) model of SRL. These strategies facilitate critical skills, such as goal-setting, planning, and self-assessment, which enhance writing outcomes. Writing self-efficacy, similarly, played a crucial role as a mediator. This finding aligns with those of prior studies, which emphasize its influence on learners’ motivation and persistence in challenging tasks (Pajares, 2003; Schunk & Zimmerman, 2007).

The findings also contribute to the broader literature by contextualizing these theoretical constructs in academic writing. While SRL research traditionally focuses on broader academic behaviors, this study demonstrates the relevance of SRL strategies to the nuanced domain of writing, an area that requires both cognitive and metacognitive engagement. The mediating role of writing self-efficacy further underscores the importance of SRL, as it bridges feedback perception and self-regulated writing ability, demonstrating the interplay between external (feedback) and internal (writing self-efficacy) factors in the writing process.

Contrary to expectations, writing feedback perception and writing self-efficacy did not directly predict writing achievement. These non-significant findings diverge from prior research, suggesting their importance (Hillocks, 2009; Magno & Amarles, 2011). Possible explanations are the variability in feedback quality, timing, or format of feedback, as well as individual differences in how learners internalize and apply feedback. Additionally, cultural factors in the South Korean context, such as a stronger emphasis on instructor authority or test-oriented learning, might have influenced learners’ perceptions and responses to feedback. Future studies should investigate whether the cultural context mediates the relationship between feedback perception and writing outcomes.

### 5.3. Practical Implications

This study’s findings have important implications for teaching practice and instructional design. Instructors should prioritize interventions that simultaneously enhance self-regulated learning strategies and writing self-efficacy to promote effective writing outcomes. Implementing structured feedback systems that provide actionable and timely insights is essential. For example, formative feedback focusing on goal-setting, reflection, and metacognitive strategies could help learners develop SRL skills. Additionally, peer feedback and self-assessment activities can encourage learners to take a more active role in the writing process, fostering self-regulation and reflective practices. Writing interventions should also aim to build learners’ confidence in writing through scaffolding and constructive feedback. To reduce writing anxiety and boost writing self-efficacy, learners should be exposed to successful writing experiences, such as completing manageable tasks or analyzing exemplary work. Furthermore, incorporating tools like reflective journals, writing portfolios, and workshops can help students internalize feedback, monitor their progress, and develop a deeper understanding of their writing journey.

### 5.4. Limitations and Future Directions

This study has some limitations that should be addressed in future research.

First, the sample was drawn from undergraduate students at a large university in South Korea, which may limit the generalizability of the findings. The sample predominantly represents a specific demographic and cultural context, potentially narrowing the scope of applicability to other populations. For instance, writing feedback perception and self-regulated learning strategies may vary significantly in more heterogeneous populations, such as those from different cultural backgrounds, educational systems, or contexts. This limitation highlights the need for future studies to incorporate more diverse academic disciplines, institutions, and cultural contexts to validate the findings across broader populations. Exploring how socio-cultural factors, such as cultural attitudes toward feedback or differing educational practices, influence self-regulation and writing outcomes could provide valuable insights into the generalizability of the results. Such studies could also identify unique factors that contribute to writing development in varying contexts, enhancing the applicability and impact of this research.

Second, self-regulated writing ability, used as a dependent variable in this study, might not have fully captured writing achievement or overall writing competence. Self-regulated writing ability is just one aspect of writing achievement, and additional metrics or broader indicators of writing success could provide a more comprehensive understanding. Additionally, if the instruments used to measure self-regulated writing ability were adapted for this study’s context, any limitations related to their cultural appropriateness or scope should be recognized. Future research could explore the validity and reliability of these instruments across diverse cultural and educational settings to ensure their applicability and relevance.

Third, the use of self-reported data introduces the possibility of biases, including social desirability and inaccuracies in self-assessment. These biases may affect the validity of the findings by overestimating or underestimating participants’ actual writing abilities and self-regulation practices. To address these limitations, future research should incorporate alternative evaluation methods that provide more objective and triangulated measures of writing achievement. For example, rubric-based assessments conducted by independent evaluators, behavioral observations of writing processes, or peer assessments could offer a more comprehensive and reliable understanding of participants’ writing abilities. Additionally, longitudinal data collection could capture developmental changes in writing achievement and self-regulated learning strategies over time, further strengthening the validity of the findings. Moreover, if the instruments used in this study were adapted for its specific context, their cultural appropriateness and scope should be considered. Future studies could validate these instruments in diverse cultural and educational settings to ensure their relevance and applicability across different populations.

Fourth, the cross-sectional nature of this study precludes causal inferences, as it captures relationships among variables at a single point in time rather than across different stages of development. Consequently, although the mediation analysis suggests potential pathways linking writing feedback perception to self-regulated writing ability through writing self-efficacy and SRL strategies, the lack of temporal data limits the ability to confirm these pathways as causal. This limitation may have substantial implications for the study’s findings, as the observed associations may be influenced by unmeasured confounding variables or reverse causality. For example, higher writing self-efficacy might not only be a result of effective feedback perception but could also influence how students perceive and utilize feedback. To address this limitation, future longitudinal studies could track changes in writing self-efficacy and SRL strategies over time to establish their developmental trajectories. Such designs would provide a stronger basis for identifying causal relationships. Additionally, experimental or quasi-experimental designs are needed to test the effects of specific feedback interventions, such as the timing, format, and content of feedback, on writing outcomes. These approaches would help confirm the causal pathways suggested by this study and offer practical insights into how feedback can be optimized to enhance self-regulated writing ability.

Fifth, this study focused on the mediating effects of writing self-efficacy and SRL strategies but did not fully account for other potential mediators or moderators. Such variables as prior writing experience, motivation, and cognitive abilities may significantly influence the relationships studied. Future research should investigate additional variables and complex psychological mechanisms that may influence these relationships to offer a more nuanced view of how feedback perception impacts self-regulated writing ability.

Sixth, the technological and contextual factors associated with this study may also pose limitations that warrant consideration. For instance, the surveys were administered online, which might have influenced participant responses or data quality. Online surveys can be subject to various challenges, such as participant distraction, lack of oversight, or technical issues that may impact the reliability and consistency of responses. Additionally, although the anonymity provided by online administration could have reduced social desirability bias, it may also have introduced variability in how participants interpreted or engaged with the survey items. Future research should explore the potential impact of survey administration methods on data quality and consider employing alternative approaches, such as in-person assessments, hybrid formats, or the use of real-time monitoring tools to ensure data accuracy. By addressing these technological and contextual factors, future studies could further enhance the robustness and validity of the findings.

Finally, the findings of this study are based on quantitative data. Incorporating both quantitative and qualitative data could provide a more robust and detailed understanding of the phenomena studied. Therefore, future research should employ a mixed-methods design to offer stronger and more concrete evidence.

Taken together, these findings underscore the importance of fostering SRL strategies and self-efficacy in writing instruction. To improve writing achievement, instructors should provide constructive and actionable feedback, encourage reflective practices, and create supportive learning environments that empower learners to actively engage in the writing process. A multifaceted approach integrating both feedback and SRL strategies would better equip learners to navigate the complexities of academic writing.

## 6. Conclusions

This study’s findings indicate that writing feedback perception positively impacts writing self-efficacy and SRL strategies, which, in turn, have a greater positive impact on self-regulated writing ability. Additionally, writing self-efficacy and SRL strategies serve as mediators between writing feedback perception and self-regulated writing ability, respectively. Therefore, to enhance undergraduates’ writing competencies and improve their writing achievement, it is necessary to provide constructive and productive positive feedback to improve their perceptions of writing feedback, implement instructor interventions that increase writing self-efficacy, and strengthen their use of SRL strategies. Overall, the findings highlight the general importance of writing feedback perception and self-regulated writing ability among undergraduates and contribute to the body of research on associated factors.

## Figures and Tables

**Table 1:** Participants.

Subjects			Phase 1 (*N* = 196)		Phase 2 (*N* = 306)	
			Frequency	%	Frequency	%
Sex	Men		84	42.86	123	40.2
	Women		112	57.14	183	59.8
Major	Engineering	Mechanical information	29	14.8	39	12.75
		Software convergence	22	11.22	31	10.13
		Architecture	20	10.2	28	9.15
		Biochemical engineering	10	5.1	20	6.54
		Electronics and electrical convergence engineering	10	5.1	16	5.23
		Sub-total	91	46.42	134	43.8
	Arts	Design convergence	54	27.56	88	28.75
		Media and animation	17	8.67	28	9.15
	Others	Major deferred (campus free major)	34	17.35	56	18.3
		Sub-total	105	53.58	172	56.2
Total			196	100	306	100

**Table 2:** Descriptive statistics and correlation analysis results.

	Phase 1 (*N* = 196)					Phase 2 (*N* = 306)			
	1	2	3	4	5	1	2	3	4
1. Writing feedback perception	-					-			
2. Writing self-efficacy	0.376 **	-				0.432 **	-		
3. Self-regulated learning strategies	0.492 **	0.533 **	-			0.510 **	0.553 **	-	
4. Self-regulated writing ability	0.322 **	0.518 **	0.567 **	-		0.393 **	0.552 **	0.648 **	-
5. Report scores	0.151 *	0.184 *	0.154 *	0.008	-	-	-	-	-
M	4.179	3.99	3.7522	3.829	81.685	4.156	3.901	3.754	3.799
SD	0.649	0.658	0.486	0.468	10.668	0.646	0.655	0.506	0.489
min.	2.67	2	2.53	2.75	65	2	2	2.1	2.42
max.	5	5	4.9	5	95	5	5	4.9	5
skewness	−0.347	−0.324	−0.037	0.206	−0.293	−0.338	−0.192	−0.283	0.119
kurtosis	−0.835	−0.26	−0.253	0.038	−1.14	−0.587	−0.381	0.065	−0.067

* *p* < 0.05, ** *p* < 0.01.

**Table 3:** Regression coefficients of predictor variables for report scores.

Variables	Unstandardized Coefficients		Standardized Coefficients	*t*	*p*	Multicollinearity	
	B	SE	*β*			Tolerance	VIF
(Constant)	69.924	7.388		9.465	0		
Writing feedback perception	0.891	1.405	0.055	0.634	0.527	0.728	1.373
Writing self-efficacy	2.378	1.535	0.147	1.549	0.123	0.602	1.661
Self-regulated learning strategies	4.724	2.3	0.214	2.054 *	0.041	0.498	2.008
Self-regulated writing ability	−5.035	2.264	−0.222	−2.224 *	0.027	0.542	1.846

* *p* < 0.05.

**Table 4:** Predicting successful writing achievement using logistic regression analysis.

Variables	B	SE	Wald	*p*
(Constant)	−0.799	1.679	0.227	0.634
Writing feedback perception	−0.104	0.321	0.105	0.746
Writing self-efficacy	0.209	0.352	0.351	0.554
Self-regulated learning strategies	1.16	0.559	4.31	0.038 *
Self-regulated writing ability	−1.329	0.556	5.717	0.017 *

* *p* < 0.05.

**Table 5:** Regression analysis results of writing feedback perception on writing self-efficacy and self-regulated learning strategies.

Predictor Variable	Criterion Variable	Unstandardized Coefficient		Standardized Coefficient	*t*	*p*
		B	SD	*β*		
Writing feedback perception	Writing self-efficacy	0.438	0.052	0.432	8.351 ***	0.000
*F* = 69.732, *R*^2^(adj. *R*^2^) = 0.187 (0.184)						
Writing feedback perception	Self-regulated learning strategies	0.400	0.039	0.510	10.345 ***	0.000
*F* = 107.023, *R*^2^(adj. *R*^2^) = 0.260 (0.258)						

*** *p* < 0.001.

**Table 6:** Multiple regression analysis results of writing feedback perception, writing self-efficacy, self-regulated learning strategies, and self-regulated writing ability.

Predictor Variable	Unstandardized Coefficient		Standardized Coefficient	*t*	*p*	VIF
	B	SD	*β*			
Constant	1.165	0.168		6.921	0.000	
Writing feedback perception	0.022	0.038	0.029	0.583	0.561	1.414
Writing self-efficacy	0.204	0.038	0.273	5.326	0.000	1.507
Self-regulated learning strategies	0.466	0.052	0.482	8.981	0.000	1.658
Criterion variable: Self-regulated writing ability, *F* = 90.906 ***, *R*^2^ (adj. *R*^2^) = 0.475 (0.469)						

*** *p* < 0.001.

**Table 7:** Bootstrapping results for the mediation effects of writing self-efficacy and self-regulated learning strategies on the relationship between writing feedback perception and self-regulated writing ability.

Mediation Path	Effect	Boot SE	Boot 95% CI	
			LLCI ^†^	ULCI ^‡^
Writing feedback perception → writing self-efficacy → self-regulated writing ability	0.154	0.028	0.102	0.211
Writing feedback perception → self-regulated learning strategies → self-regulated writing ability	0.234	0.036	0.165	0.304

^†^ LLCI = boot lower limit value within the 95% confidence interval of the bootstrapped indirect effect; ^‡^ ULCI = boot upper limit value within the 95% confidence interval of the bootstrapped indirect effect.

## Data Availability

Data are available from the corresponding author upon reasonable request.
